# Effect of the roasting levels of *Coffea arabica* L. extracts on their potential antioxidant capacity and antiproliferative activity in human prostate cancer cells

**DOI:** 10.1039/d0ra01179g

**Published:** 2020-08-17

**Authors:** Laurent dos Santos de Souza, Isabella Porto Carrero Horta, Lana de Souza Rosa, Larissa Gabrielly Barbosa Lima, Jeane Santos da Rosa, Julia Montenegro, Lauriza da Silva Santos, Raquel Bernardo Nana de Castro, Otniel Freitas-Silva, Anderson Junger Teodoro

**Affiliations:** Laboratory of Functional Foods, Universidade Federal do Estado do Rio de Janeiro Rio de Janeiro 22290-240 Brazil laurentkio05@gmail.com isapch@hotmail.com atteodoro@gmail.com; Empresa Brasileira de Pesquisa Agropecuária, Embrapa Agroindústria de Alimentos Rio de Janeiro 23020-470 Brazil jeane.rosa@embrapa.br otniel.freitas@embrapa.br

## Abstract

Coffee, besides being one of the most consumed stimulating beverages in the world, has important bioactive activities, which have been attracting increasing attention from researchers. However, the standard process of roasting causes changes in its chemical composition. In the present study, extracts obtained from green and roasted beans (light, medium and dark) of *Coffea arabica* Linnaeus were submitted to high-power ultrasonic extraction and atomization by spray drying. Colorimetric analysis was used to classify the roasting levels of the dried extract samples. The effects of the roasting process on the bioactivity of the dried extracts were verified through the following assays: caffeine, chlorogenic acid and caffeic acid, by HPLC-PDA; total phenolics by Folin–Ciocalteu; antioxidant activity by DPPH, FRAP, ABTS and ORAC; antiproliferative activity, using the MTT assay; and cell cycle and apoptosis by flow cytometry in metastatic prostate cancer cell lines from bone (PC-3) and brain (DU-145). The results showed that the lowest levels of caffeine, chlorogenic and caffeic acids were observed in dark roasted coffee. In comparison to medium and dark extracts in PC-3 cells, the green and light coffee extracts had higher antioxidant activities and promoted cytotoxicity followed by cell cycle arrest in phase S and apoptosis induction. Thus, the roasting level of the coffee extracts may be related to the potential chemoprotective effects of *Coffea arabica* L. in prostate cancer cells.

## Abstract 2

Coffee, besides being one of the most consumed stimulating beverages in the world, has important bioactive activities, which have been attracting increasing attention from researchers.

## Graphical abstract

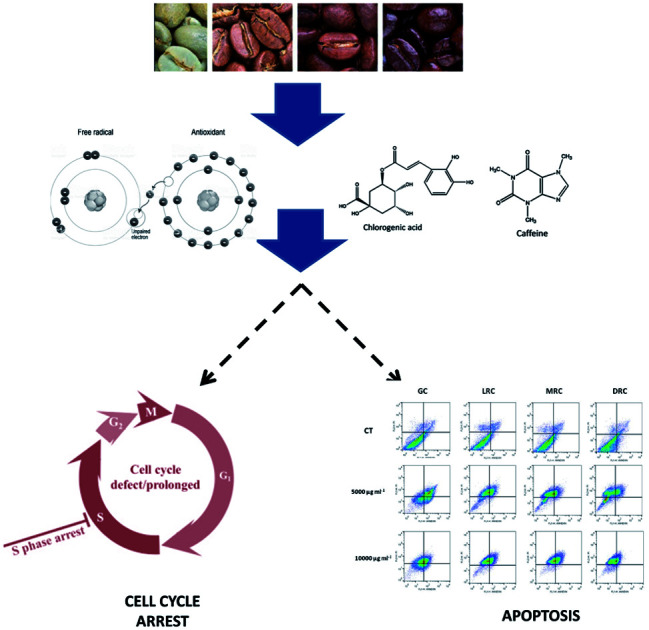

## 1. Introduction

Despite the emergence of “energy drinks”, coffee is still the main beverage used to start and continue daily activities,^1^ due to its stimulant compounds and phytochemicals,^78^ which besides improving performance, can also promote wellness.^2^ Brazil is the world's leading producer and exporter of coffee, with ten producing states. Minas Gerais is the state with the highest production, followed by Espírito Santo, while Rio de Janeiro occupies the seventh place. The countries that most consume coffee are the United States, followed by Germany, Italy, Japan and Belgium.^3^

Coffee belongs to the Rubiaceae family and Coffea genus, which has more than 100 species and subspecies, 25 of which are marketed.^4^ Brazil is the main producer of the *C. arabica* and *C. canephora* species.^5^ The reasons for high consumption of this beverage are due to its palatability and economic accessibility.^6^ Arabica coffee accounts for 70% of the world's exports and its sensorial characteristics are considered more appealing than *C. canephora*.^7^ However, the beverage from *C. canephora* contains higher levels of bioactive compounds, such as chlorogenic acids, trigonelline, caffeic acid and caffeine among others.^8^

Coffee displays the highest antioxidant potential compared to other popular beverages.^10^ The degree of coffee roasting is controlled by the combination of time and temperature. It is usually qualitatively assessed by colour and classified as light, medium or dark roasted coffee. The roasting process causes changes in the chemical composition and biological activity of coffee: while natural phenolic compounds may be lost, other antioxidant compounds are formed, such some Maillard reaction products. Despite the many studies addressing the antioxidant activity of roasted coffee, little has been reported about the biological activity and variations in the composition of the product in different phases of the roasting process.^11,12^

In Brazil, chronic non-communicable diseases (NCDs) are the main cause of death in adults and the elderly.^9^ NCDs are associated with endogenous factors such as genetic predisposition and exogenous factors like inadequate eating habits.^13^ An analysis conducted by the Brazilian Institute of Geography and Statistics (IBGE) showed that the pattern of food consumption among the Brazilian population has increased the demand for foods with high caloric densities and low nutritional content.^14^ Therefore, a good public health strategy would be to encourage consumption of less ultra-processed foods. Although, there is a change in this pattern of eating, natural foods have always been present in the Brazilian diet. Regular coffee consumption, for example, is a habit that has not changed. In fact, there was an increase in this consumer behavior of approximately 5% in 2018, according to studies of the Brazilian research coffee consortium.^15–17^ This consortium is a strategic arrangement with more than 40 Brazilian institutions, which aims to develop technologies for all stages of the coffee production chain. The consortium is organized by the Brazilian Agricultural Research Corporation – Embrapa, linked to the Ministry of Agriculture. Thus, Brazil is not only the largest coffee producer and exporter worldwide but also a global leader in technical and scientific research on coffee.^80^

Prostate Cancer (PCa) is one of the most prevalent cancer types throughout the world, and is one of the most intensely studied problems involving human disease. Several cancer cell lines have been developed and used to determine the mechanisms underlying cancer tumorigenesis and identify markers of therapeutic response. Cancer cell lines have several advantages, including infinite proliferation ability and amenability to high-throughput drug screening. Along with LNCaP and PC3 cells lines, DU-145 cells were once considered part of the triad that constituted the gold standard of PCa cell culture lines. DU-145 cells were first isolated from a brain metastatic prostate tumour. PC3 cells were isolated from a vertebral metastatic prostate tumour and are similar to DU-145 cells in that they are hormone-insensitive and have no androgen receptors.^18–20^

The objective of the present work was to analyse the antioxidant activities and the effects of green and roasted solid (spray drying) coffee extracts at three gradient levels (light, medium and dark) in prostate adenocarcinoma cell lines (PC-3 and DU-145).

## 2. Materials and methods

### 2.1 Samples

*Coffea arabica* (variety ‘Catuaí vermelho-amarelo’) was grown in Bom Jardim (Brazil). The green coffee bean samples were stored in the Laboratory of Molecular Diagnosis and Mycology of Embrapa Food Technology located in the state of Rio de Janeiro, Brazil.

### 2.2 Raw material and production of extracts

The steps to obtain the coffee extracts started with manual sorting to discard deficient beans and dirt, to select those with good quality and to keep the samples homogeneous.^21^

**Fig. 1:**
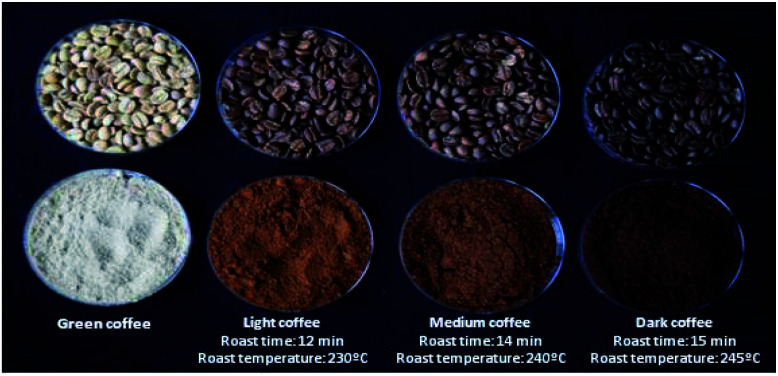
Green and roasted coffee beans and their respective ground beans. Roasting time and temperature according to each type.

In industry, hot air roasting is the most widespread technology used. Medium-dark roasts are the most widely sold in Brazil.^22^ The beans were divided into four portions: one for green coffee extracts, and the others according to roast processing, namely light roasting (12 minutes at 230 °C), medium (14 minutes at 240 °C) and dark (15 minutes at 245 °C). The beans were roasted in a Gene Café® roaster (CBF-101, Kyungki-Do, Korea) and were classified according to the Agtron Scale and the manufacturer's manual^23^ (Fig. 1).

The green coffee beans were ground in the disc mill (Laboratory Mill 3600) by the grain stiffness criterion. After natural cooling, the roasted beans were further ground by brushed steel grinder (Cuisinart®, East Windsor, NJ, USA) to fine consistency. Then, the grain sizes of the samples were standardized using two sets of sieves (Bertel, São Paulo, Brazil) with 0.850 and 0.600 mm mesh.

After obtaining granulated green and roasted coffee, infusions were carried out to obtain the extract. In a 250 mL beaker, 25 g of the powder was solubilized in 100 mL of distilled water at 90–95 °C for 5 minutes. Afterwards, these infusions were cooled in an ice bath and placed in a high-power ultrasonic processor (Hielscher® UIP1000hdT, Teltow, Germany) (350 W; amplitude = 70% for 10 min). Then the samples were transferred to Falcon tubes and centrifuged at 7000 rpm for 14 min. The liquid part (supernatant) was transferred to a 200 mL beaker and the solid part (precipitate) was discarded. The supernatant was submitted to analysis of total solids (g 100 g^−1^) with a refractometer (Atago PAL-Alpha digital). This measurement was used to delineate the feasibility of spray drying (dehydration at 180 °C; vacuum of −60 mbar). After drying, the extracts were stored individually in light-protected zipped laminated bags in a cooling chamber at −80 °C until analysis.

### 2.3 Colorimetric analysis

The colorimetric analysis of the samples was performed using a Konica Minolta CM-5 benchtop colorimeter (Sensing Americas, Inc.) with prior calibration. Three replications were performed, with direct reading of reflectance. The standard colour scale used was CIELAB, with the coordinates L*, a*, b*, where L* is lightness, a* is red/green intensity and b* is yellow/blue intensity.^24^ Results were expressed as mean of replicates plus or minus standard deviation.

### 2.4 Characterization of dry extracts by HPLC

#### 2.4.1 Determination of caffeine

The caffeine content was measured by HPLC,^25^ with a Waters Alliance 2695 photodiode array detector (PDA), 2996 chromatograph and Empower® software (Waters, Milford, MA, USA), together with a Hypersil C_18_ BDS column (5 cm × 4.6 mm and 2.6 μm – Thermo Scientific, Massachusetts, USA). The mobile phase was composed of 10% acetonitrile in 0.5% (v/v) acetic acid solution. Samples were submitted to extraction with 1 g to 10 mL of the mobile phase for 10 minutes in a 25 mL volumetric flask with an ultrasonic bath. Each sample was filtered through filter paper (rapid filtration type) and microfiltered into disposable hydrophilic Teflon filter units with porosity of 0.22 μm. The concentration of the caffeine external standard was 1 mg mL^−1^, solubilized with the mobile phase. The wavelength was 280 nm with flow rate of 0.5 mL min^−1^. The injection volume was 20 μL and the result was expressed as g of caffeine per 100 g of sample (% w:w).

#### 2.4.2 Determination of chlorogenic acid and caffeic acid

The levels of chlorogenic acid (5-CQA) and caffeic acid were determined by the HPLC method described by Trugo and Macrae (1984),^26,27^ using a Waters Alliance 2695 chromatograph, 2996 PDA and Empower® software (Waters, Milford, MA, USA), along with a Hypersil C_18_ BDS column (5 cm × 4.6 mm and 2.6 μm; Thermo Scientific, Milford, MA, USA). The mobile phase gradient consisted of the initial composition of 5% methanol (phase A) and 95% of 0.1% formic acid solution (phase B), maintained for 6 min. After 8 minutes, the composition of the mobile phase reached 80% of phase A and remained there for up to 10 min. After 11 min, the composition reached 100% of phase A, and from 12 min to 15 min, the composition returned to the initial conditions. Detection was performed at 325 nm. The flow rate of the mobile phase was 1 mL min^−1^ and the injection volume was 3 μL. Extraction was performed in an ultrasonic bath for 20 min with 1 g of the sample in 5 mL of acetonitrile (20%) in ultrapure water (v/v), in a 25 mL volumetric flask. The samples were then centrifuged and microfiltered in disposable hydrophilic Teflon® filter units with porosity of 0.22 μm. A pre-calibrated system with chlorogenic acid and caffeic acid external standards (Sigma-Aldrich, New York, NY, USA) at 1.2 mg mL^−1^ concentration in 0.5% formic acid in ultrapure water was used. Results were expressed as g of the analytes per 100 g of sample (% w/w).

### 2.5 Determination of total phenolic compounds

An initial aqueous solution (50 mg/10 mL) of each soluble dried Arabica coffee extract was prepared. Then, three dilutions of these solutions, with aliquots of 10, 50 and 100 μL, were added to 2 mL of ultrapure water (these solutions also were used for measurement of antioxidant activity, except ORAC).

The total phenolic content of the extracts was determined according to the Folin–Ciocalteu method, as described by Hudáková *et al.*^28^ From each of the dilutions, in triplicate, 500 μL was retrieved and added to 2.5 mL of Folin–Ciocalteu reagent and 2.0 mL of 4% sodium carbonate solution. The mixture was allowed to rest for 2 h in the dark. Measurements were performed at 750 nm in triplicate, in a Shimadzu UV-VIS 2700 spectrophotometer (Nakagyo-ku, Kyoto, Japan). Gallic acid, in the concentration range of 0–100 mg mL^−1^, was used for the calibration curve. The concentration of total phenolic compounds of the extracts was expressed as gallic acid equivalents, which reflect the phenolic content as the amount of gallic acid in mg 100 g^−1^ dry weight of the samples.

### 2.6 Determination of antioxidant activity

The analysis of the antioxidant activities of the soluble extracts of green Arabica coffee and their respective roasted samples were evaluated through four different methods: DPPH scavenging activity or ABTS radical cation scavenging activity; ferric reducing antioxidant power (FRAP); and oxygen radical absorbance capacity (ORAC).^27^

#### 2.6.1 Evaluation of the sequestering activity of DPPH radicals

The diluted extracts were mixed with 2.5 mL of DPPH methanolic solution (0.06 mM) and allowed to react for 1 h in the dark. Measurements were performed at 515 nm with a Shimadzu UV-VIS 2700 spectrophotometer (Nakagyo-ku, Kyoto, Japan). The analysis was performed in triplicate. The decline in the DPPH radical absorbance concentration caused by the extracts was compared to a Trolox standard. The results were expressed as μmol Trolox equivalent (TE) g^−1^ dry weight of the samples.^29,30^

#### 2.6.2 Total antioxidant activity analysis by ABTS*+ radical capture

ABTS cations were prepared by mixing an ABTS stock solution (7 mM in water) with 2.45 mM of potassium persulfate. This mixture was allowed to stand for 24 h at room temperature until the reaction was completed and the absorbance was stable. The antioxidant capacity assay was carried out by the ABTS/TEAC method created in 1993 by Miller *et al.*^31,32^ The ABTS solution (2.5 mL) was added to diluted sample extracts, or commercial antioxidant Trolox to obtain the calibration curve (Trolox equivalent – TEAC), and mixed thoroughly. Absorbance was measured at 734 nm after 6 min. Results were expressed as μmol TE g^−1^ dry weight of the samples.

#### 2.6.3 Determination of the total antioxidant activity by the iron reduction method (FRAP)

The extracts were measured for antioxidant activity by FRAP according to Rufino *et al.*^27^ Aliquots of 2.7 mL of TPTZ reagent (ferric-2,4,6-tripyridyl-*s*-triazine) were mixed with the diluted extract samples. After 30 min at 37 °C, the absorbance was read at 595 nm. The antioxidant capacity was expressed as ferrous sulphate equivalents (μmol FeSO_4_ g^−1^ dry weight of the samples).^33^

#### 2.6.4 Antioxidant activity test by the ORAC method

The ORAC assay was conducted with an automatic plate reader (SpectraMax i3x, San Jose, CA, USA) with 96-well plates. Analysis was conducted with eight coffee extract dilutions (0.005, 0.0010, 0.0025, 0.0050, 0.01, 0.02, 0.03 and 0.04 μg mL^−1^), after which 120 μL of the fluorescein solution and 60 μL of 2,2′-azobis(2-amidino-propane) dihydrochloride (AAPH) was added.^34^

Analysis was conducted in phosphate buffer with pH 7.4 at 37 °C. Peroxyl radical was generated using the AAPH reagent, which was freshly prepared for each run. Fluorescein was used as the substrate. Fluorescence conditions were as follows: excitation at 485 nm and emission at 520 nm. The standard curve was linear between 1 μM and 90 μM Trolox. Results were expressed as μmol of TE g^−1^ dry weight of the samples of coffee extract.

### 2.7 Antiproliferative activity of coffee extracts

#### 2.7.1 Reagents

Dimethyl sulfoxide (DMSO), 3-(4,5-dimethylthiazol-2-yl)-2,5-diphenylthiazolium bromide (MTT), RPMI 1640 medium, propidium iodide (PI) and RNase A were purchased from Sigma Chemical Co. (St. Louis, MO). The Annexin V-FITC apoptosis detection kit was acquired from eBioscience (San Diego, CA). An antibiotic solution (penicillin–streptomycin), nonessential amino acids, *N*-2-hydroxyethylpiperazine-*N*-2-ethanesulfonic acid (HEPES), phosphate buffered solution (PBS) and trypsin–EDTA solution (2.5 g L^−1^ trypsin and 0.2 g L^−1^ EDTA) were obtained from Gibco (Scotland, UK).

#### 2.7.2 Cell culture and treatments

Human prostate cancer cell lines (DU-145 and PC-3) were obtained from the Cell Bank of Rio de Janeiro Federal University. Cell culture was carried out in culture flasks in RPMI 1640 medium (Sigma, New York, NY, USA) supplemented with 10% foetal bovine serum (FBS), 1% penicillin and streptomycin (PS), pH 7.4, under an atmosphere of 5% CO_2_ at 37 °C. The cells, when at 70–80% confluence, underwent trypsinization, about twice a week. For each experiment, cells were plated at concentrations of 2.0 × 10^5^ in 6-well plate for cell cycle and apoptosis, and 4.0 × 10^4^ cells cm^−2^ in 96-well microplate for cell viability analysis.^27^ A control group was included in all the tests, treated only with culture maintenance medium, free of the samples. All experiments were performed in triplicate.

#### 2.7.3 Cell viability assay

The viability of the cells was analysed by measuring the enzymatic activity of their mitochondria by the MTT (3-[4,5-dimethyl-thiazol-2-yl]-2,5-diphenyl-tetrazolium bromide) assay, as described by Mosmann (1983).^35^ 2.0 × 10^4^ cells per well were plated in 96-well plates. After 24 hours, they were incubated with the soluble coffee extracts in concentrations ranging from 78.1 to 10 000 μg mL^−1^. After 24 hours of treatment, the medium was changed in all wells (150 μL) and 10 μL of MTT (5 g L^−1^) was added to each well. After 4 h of incubation with the MTT salt, 80 μL from each well was collected and placed in an oven and 50 μL per well of dimethyl sulfoxide (DMSO) was added to dissolve the generated product. After 15 minutes in the oven, the plate was read with a microplate reader (Polaris, CELER®) at 570 nm. The cell proliferation inhibition rate (CPIR) was calculated as a percentage based in on the formula:CPIR = (1 − mean value of the experimental group/mean value of the control group) × 100.

#### 2.7.4 Cell cycle analysis

For cell cycle assay, 4.0 × 10^4^ cells per well were plated in 6-well plates and were incubated with extracts for 24 h. Cells were rinsed briefly with calcium and magnesium-free phosphate-buffered saline (PBS) and detached with trypsin at room temperature. After centrifugation, the cells were washed twice with phosphate-buffered saline; and then resuspended in 500 μL of ice-cold Vindelov solution^36^ containing 0.1% Triton X-100, 0.1% citrate buffer, 0.1 mg mL^−1^ RNase, and 50 mg mL^−1^ propidium iodide (Sigma Chemical Co., St. Louis, MO, USA). After incubation for 15 min, the cell suspension was analysed for DNA content by flow cytometry using a FACSCalibur flow cytometer (Becton Dickinson, Mountain View, CA, USA). The relative proportions of cells with DNA content indicative of apoptosis (<2n), G_0_:G_1_ diploid (2n), S (2n < phase < 4n), and G_2_:M phase (4n) were obtained and analysed using the CellQuest WinMDI 2.9 program.

The percentage of cell population at a particular phase was estimated with the FlowJo software. The cell dissociation procedure did not affect fluorescence under the experimental conditions used in this study, or in any other studies of which we are aware. Nuclei of viable cells were gated according to the FL2-width-to-FL2-area ratio.

#### 2.7.5 Detection of apoptosis by annexin V-FITC

To measure the rate of apoptosis, 4.0 × 10^4^ cells per well were plated in 6-well plates and were incubated with extracts for 24 h. Then, the cells were subjected to staining with annexin V conjugated with FITC. The non-adherent cells were collected, and adherent cells were quickly washed with a calcium/magnesium-free buffered saline solution (BSS) and were detached with 0.125% trypsin/EDTA (Sigma Chemical Co., St. Louis, MO, USA) at room temperature. Subsequently, apoptotic and necrotic cells were stained with annexin V FITC/propidium iodide (PI) (BD PharMingen, Mountain View, CA, USA) according to the manufacturer's instructions, quantified with a flow cytometer (FACSCalibur, BD Bioscience, Mountain View, CA, USA), and analysed using two specific programs, Cell Quest and FlowJo.

### 2.8 Statistical analysis

Results are presented as mean with the corresponding standard deviation of triplicates of two different experiments (*n* = 6). Data were analysed with the statistical software GraphPad Prism (version 7.0, GraphPad Software, San Diego, CA, USA). One-way analysis of variance (ANOVA) and the Tukey post-hoc test at a confidence level of 95% were used to test cell viability, cell cycle, and apoptosis.

## 3. Results and discussion

### 3.1 Physicochemical results

#### 3.1.1 Colorimetry

The colorimetric results of the samples, as expected, were different from each other and similar to the data obtained in the literature.^23^ The CIELAB colour scale has three parameters: L*, for lightness, ranging from zero (black) to 100 (white); the coordinate a*, which goes from positive (+) for the red component to negative (−) for the green component; coordinate b*, which goes from positive (+) for the yellow component and negative (−) for the blue component. The green coffee extract, as expected,^37^ presented higher luminosity (L*) value, which was inversely proportional to the degree of roasting, but there were no significant differences between the soluble extracts of light and medium roasted Arabica coffee. The soluble extract of green coffee showed a greenish-yellow coloration, and the roasted ones showed an initial red-yellow coloration, quickly turning bluish red (Table 1).

**Table 1:** Colorimetric coordinates (L*, a*, b*) of the dry extracts of Arabica coffee^a^

Coordinates	GC	LRC	MRC	DRC
L*	78.71 ± 0.07^a^	56.85 ± 0.05^b^	55.47 ± 2.38^b^	50.42 ± 0.02^c^
a*	−0.44 ± 0.02^a^	11.26 ± 0.01^b^	10.13 ± 0.01^c^	10.52 ± 0.01^d^
b*	20.45 ± 0.01^a^	33.13 ± 0.03^b^	29.13 ± 0.04^c^	28.96 ± 0.02^d^

a Results of colorimetry by the L*, a*, b* coordinates of the dried Arabica coffee extracts by atomization. Different letters mean significant differences among treatments (*p* < 0.05). Abbreviations: GC – green coffee, LRC – light roasted coffee, MRC – medium roasted coffee, DRC- dark roasted coffee.

According to De Oliveira *et al.*,^38^ in the roasting process, the decrease of luminosity is proportional to the roasting intensity due to the darkening of the beans. This occurs due to three biochemical processes: Maillard reactions, Strecker degradation and sugar caramelization.^39,40^ These exothermic reactions determine the sensory characteristics of roasted coffee beans, such as flavour, aroma and colour.

#### 3.1.2 Bioactive compounds from the extracts of green and roasted Arabica coffee

##### 3.1.2.1 Quantification of caffeine by HPLC

**Fig. 2:**
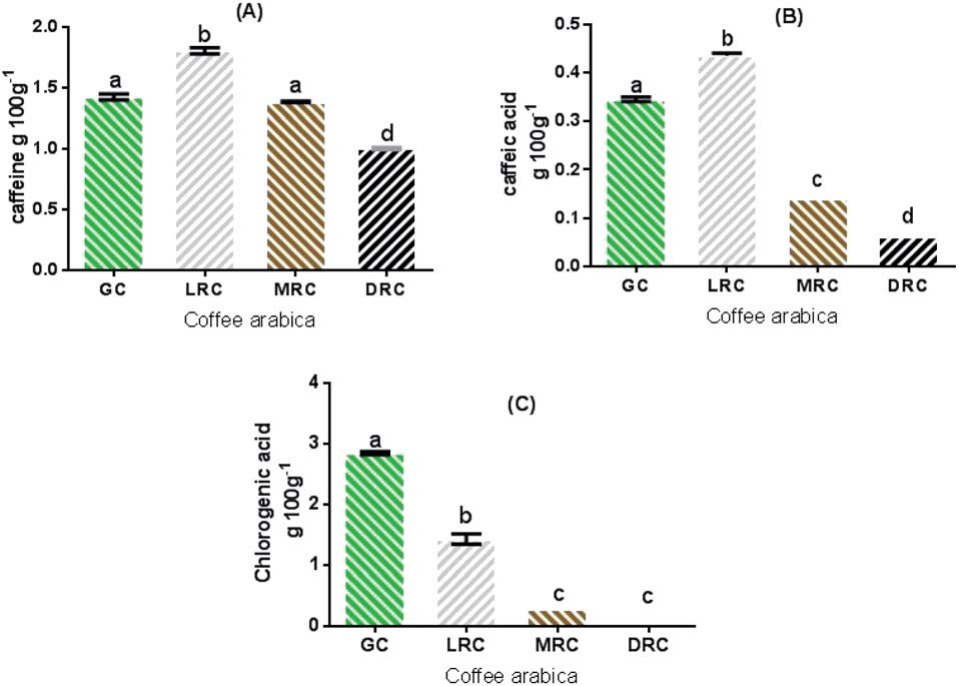
Caffeine (A), caffeic acid (B) and chlorogenic acids (C) content in the soluble extracts of Arabica coffee. Different letters mean significant differences among treatments (*p* < 0.05). Abbreviations: GC – Green Coffee, LRC – Light Roasted Coffee, MRC – Medium Roasted Coffee, DRC – Dark Roasted Coffee.

The caffeine content values in the extracts are shown in Fig. 2A. The light roast coffee had the highest concentration (1.8 ± 0.3 g/100 g). The green and medium roasted coffee samples did not present significant differences (*p* < 0.05), and the lowest caffeine content was observed in dark roasted coffee extract (1.0 ± 0.1 g/100 g).

Caffeine is a thermoresistant alkaloid that undergoes better release in aqueous media after light roasting, but may suffer partial loss by the degradation of the coffee bean mass by dehydration in the more intense roasting phase.^39,41^

##### 3.1.2.2 Quantification of chlorogenic acid and caffeic acid by HPLC

Caffeic acid is a thermosensitive bioactive of the hydroxycinnamic group of the phenolic acid family.^43^ All the extracts analysed showed significant differences (*p* < 0.05), and the highest concentration was found in the light roast coffee (0.43 ± 0.01 g/100 g). Green coffee (0.34 ± 0.007 g/100 g), medium roast (0.14 ± 0 g/100 g) and dark roast (0.06 ± 0 g/100 g) extracts presented lower values (Fig. 2B). Higher levels of caffeic acid can be produced from the final breakdown of chlorogenic acid by thermal reactions. Caffeic acid, however, is also thermally degraded as these reactions continue.^44^

Another important bioactive compound in Arabic coffee beans is chlorogenic acid, a thermosensitive phenolic acid composed by ester isomers of quinic and caffeic acid,^48,49^ which is associated with high antioxidant activity. Among the chlorogenic acid isomers found in green coffee beans, 5-CQA (neochlorogenic acid) is the most abundant, composing about 80% of chlorogenic acids, followed by 4-CQA (cryptochlorogenic acid) and 3-caffeoylquinic acid (3-CQA).^50^ Only 5-CQA was analysed in this study. Chlorogenic acid concentrations were higher in green coffee extracts (2.84 ± 0.03 g/100 g), but the extracts of the medium roasted and dark roasted beans did not present significant differences (*p* > 0.05) (Fig. 2C).

According to Manach *et al.*,^45^ the bioavailability of caffeic acids is higher than chlorogenic acid because the latter's breakdown by esterase enzymes in the intestine, increasing the content of caffeic acids.

#### 3.1.3 Total phenolic compounds in dry coffee extracts

**Fig. 3:**
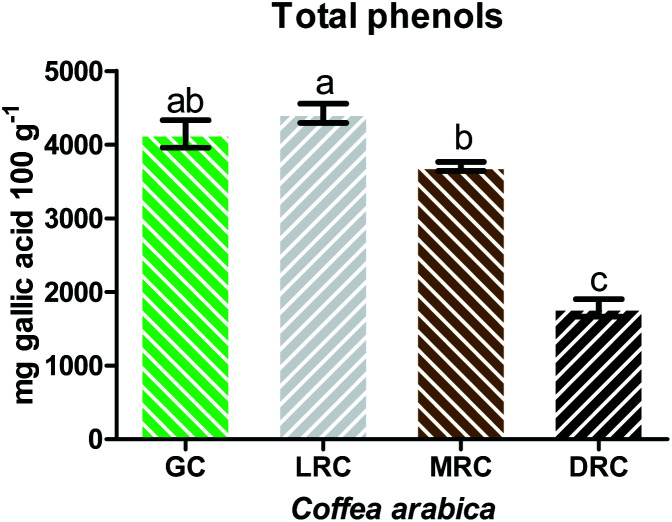
Total phenolic contents in soluble extracts of Arabica coffee. Different letters mean significant differences among treatments (*p* < 0.05). Abbreviations: GC – Green Coffee, LRC – Light Roasted Coffee, MRC – Medium Roasted Coffee, DRC- Dark Roasted Coffee.

The quantification of the total phenolic compounds of the extracts of green and roasted Arabica coffee was performed by the Folin–Ciocalteu assay.^51^ This analysis demonstrated higher concentration of total phenolics in the extract of light roasted Arabic coffee (4420.58 ± 15.25 mg gallic acid 100 g^−1^), but without significant differences with the green coffee extract (*p* > 0.05). The green coffee extract (4140.35 ± 24.53 mg gallic acid 100 g^−1^) also did not present significant differences with the medium roast, and the lowest concentration of phenolic compounds was observed in extracts of dark roasted coffee, with value of 1780.19 ± 15.52 mg gallic acid 100 g^−1^ (Fig. 3).

Total phenolic compounds increased from green to light roast, likely due to the breakdown of chlorogenic acid into caffeic acid, which has higher antioxidant capacity.^52^ The content of phenolic compounds decreased as roasting degree increased.

#### 3.1.4 Antioxidant activity of extracts of green and roasted Arabic coffee

The analysis of the antioxidant activity of soluble extracts of green coffee and roasted coffee was performed through four different methods: ORAC, ABTS, FRAP and DPPH. Due to the various types of free radicals and their different means of action in living organisms, there is no simple and universal test method that can measure the antioxidant capacity exactly and quantitatively. Hence, a single analysis would be insufficient to express antioxidant activity.^53^ The FRAP assay is based on reducing capacity. DPPH, ABTS and ORAC are based in free radical scavenging capacity, where ABTS is more hydrophilic than DPPH.^79^ ORAC is a more direct method because it is based in a naturally occurring radical, unlike DPPH and ABTS, which are stable synthetic radicals.

The most sensitive assays were DPPH and ABTS, followed by ORAC and FRAP (all extracts presented concentration dependent activity). Trolox (a vitamin E analogue) was used as the standard for measuring antioxidant strength of the samples for DPPH, ABTS and ORAC.^54,55^

The ABTS and FRAP methods showed similar results in all extracts, in accordance with the data from total phenolic analysis, they are also in line with the results of Moreira *et al.*,^56^ who correlated chlorogenic acid monomers with higher FRAP activity and the soluble compounds of caffeine and caffeic acid with superior effects in ABTS analysis.

**Fig. 4:**
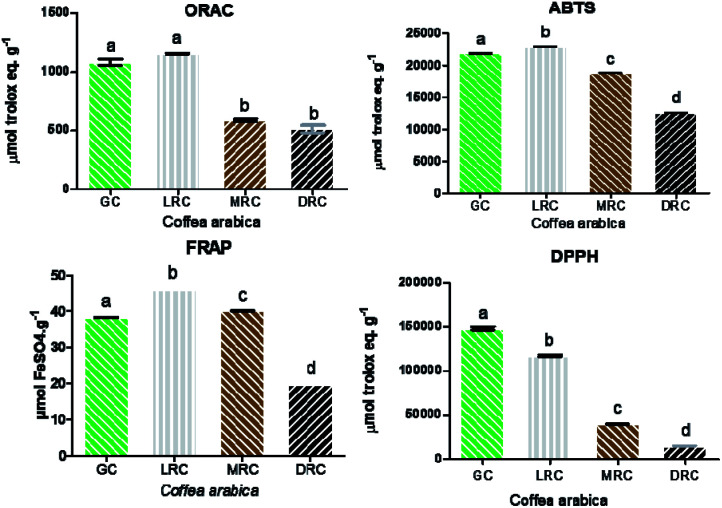
Antioxidant activity according to DPPH, ABTS, FRAP and ORAC analysis of spray-dried Arabica coffee extracts. The different letters mean no significant differences between them (*p* > 0.05). Abbreviations: GC – Green Coffee, LRC – Light Roasted Coffee, MRC – Medium Roasted Coffee, DRC – Dark Roasted Coffee.

Light roast coffee extract presented the highest antioxidant activity (22 933.62 ± 21.92 μmol TE g^−1^), followed by green coffee (21 808.91 ± 167.66 μmol TE g^−1^), medium roast (18 884.72 ± 23.18 μmol TE g^−1^) and dark roast (12 600.13 ± 24.22 μmol TE g^−1^) (Fig. 4). The results of the FRAP method followed almost the same pattern, with light roasted Arabica coffee extract having the highest activity (45.85 ± 0.01 μmol FeSO_4_ g^−1^), followed by the medium roasted coffee (40.01 ± 0.03 μmol FeSO_4_ g^−1^), green coffee (38.24 ± 0.06 μmol FeSO_4_ g^−1^) and dark roast (19.56 ± 0.01 μmol FeSO_4_ g^−1^) (Fig. 4).

Flavonoids are thermosensitive compounds that can be easily degraded even in the mildest roast.^57^ Chlorogenic acids (CGAs) are thermosensitive and also abundant in green coffee extracts. CGAs are directly related to the reduction of the DPPH radical, which is consistent with the results of the present work.^58^

Results related to extracts in the DPPH assay showed a roast-dependent decrease, which coincided with the chlorogenic acid content found in the samples.^59^ In the ORAC method, there were no significant differences (*p* > 0.05) between the green coffee and light roasted coffee extracts (1081.5 ± 27.5 and 1150.5 ± 5.16 μmol TE g^−1^) and between extracts of medium roast and dark roast coffee (584.33 ± 14.33 and 511.66 ± 33 μmol TE g^−1^ sample) (Fig. 4), indicating a negative effect of the roasting process on the antioxidant capacity.

### 3.2 Effects of green and roasted coffee extracts on cell viability, cell cycle and apoptosis of PC-3 and DU-145

**Fig. 5:**
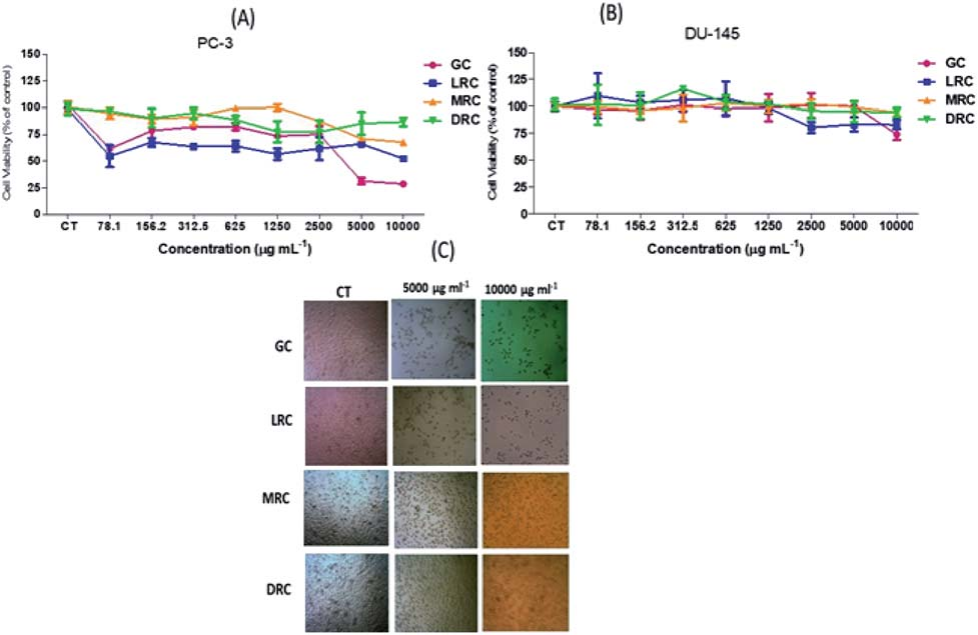
Percentage of cell viability in comparison with control of prostate tumour PC-3 (A) and DU-145 (B) cells by the MTT method, after 24 h of treatment with spray-dried aqueous coffee extracts at concentrations from 78.1 to 10 000 μg mL^−1^ (C) Photos of wells with concentrations of control, 5000 to 10 000 μg mL^−1^. Abbreviations: GC – Green Coffee, LRC – Light Roasted Coffee, MRC – Medium Roasted Coffee, DRC – Dark Roasted Coffee.

By metabolizing the salt of MTT (3-(4,5-dimethylthiazol-2-yl)-2,5-diphenyltetrazolium bromide) into formazan by the mitochondrial enzymes of PC-3 and DU-145 malignant cells, it was possible to analyse the cytotoxic effects of Arabica coffee extracts. In PC-3 cells, we observed a significant reduction of cell viability (*p* < 0.05) with a low dose of green coffee and light roasted extracts (78.1 μg mL^−1^) when compared to medium and dark roasted extracts (*p* < 0.05). Those samples reduced the viability by almost 50% and remained stable as concentration increased. However, the green Arabica coffee extract at higher concentrations (5000 μg mL^−1^ and 10 000 μg mL^−1^) decreased the cell viability of PC-3 by around 70%. The medium roasted coffee extract caused a significant reduction (*p* < 0.05) of only 33% at the highest concentration (10 000 μg mL^−1^), whereas the soluble extract of dark roasted Arabica coffee caused an average 13% reduction (Fig. 5).

No significant differences (*p* > 0.05) were observed in viability of DU-145 cells after treatment with soluble extracts of light green, medium and dark coffee extracts (*p* > 0.05). However, extracts of soluble green Arabica coffee induced the most significant (*p* < 0.05) reduction (26%) in cell viability, compared with the other extracts after treatment with the highest concentration (10 000 μg mL^−1^) (Fig. 5B). Soluble extracts of medium and dark Arabica coffee promoted a very small decrease (5%) only at the highest concentration (10 000 μg mL^−1^) (Fig. 5B), without significant differences (*p* < 0.05) compared with untreated cells.

PC-3 carcinogenic cells treated at concentrations of 5000 and 10 000 μg mL^−1^ of soluble green coffee and light roasted coffee extracts showed significant cell viability reductions compared to control cells (*p* < 0.05) (Fig. 4C).

DU145 and PC3 prostate cancer cell lines have moderate and high metastatic potential, respectively. It was reported that DU145 and PC3 cells expressed a higher number of the selected inflammatory-related genes that were upregulated in hypoxia with a more complete and persistent response in more aggressive PC3 cells.^60,61^ Our data indicate that coffee extract acts more effectively against cells with greater metastatic potential. Other researchers incubated extracts rich in chlorogenic acids of green Arabica coffee and green tea in four tumour cell lines and found that their effects did not increase the proliferation of cancer cells, negatively affecting the cellular viability and the proliferation in a dose-dependent form.^62^ Differences in extraction involving distinct solutions (organic solvent and water) produced extracts with different chlorogenic acid profiles and content, which can explain the different responses of cell lines.

Coffee constituents have been described as having genotoxic and mutagenic properties, but also antimutagenic and antioxidant activities and the capacity to inhibit the action of carcinogens. The meta-analysis of Sang *et al.*^63^ of epidemiological studies showed that coffee consumption reduces the risk of liver cancer. Previous studies have suggested the inhibitory effect of caffeine on the adhesion, motility and proliferation of DU-145 and PC-3 prostate cancer cells.^42^

Many bioactive compounds have been shown to be effective at reducing viability of cancer cell lines while not affecting non-cancer cells, which is an added advantage to their potential as natural chemopreventive substances against cancer. Several studies have demonstrated no toxic effects of coffee on non-cancer cells, such as the fibroblast CCD-18Co line, normal macrophages cells (RAW 2647) and hepatocytes o(AML-12).^62,64^ In such cases, coffee compounds display cell- and tissue-specificity. Priftis *et al.*^65^ investigated the toxicity of roasted and green coffee extracts on myoblasts and endothelial cells and found that myoblasts were more sensitive to green coffee extracts, in contrast to endothelial cells.

Based on our data of cell growth inhibition by Arabica coffee extracts in PC-3 cells, we examined the cell cycle and apoptosis by flow cytometry. Significant changes were observed compared to the control (*p* < 0.05) in PC-3 cells after treatment with high concentrations (5000 and 10 000 μg mL^−1^). Green and light Arabic coffee extract promoted a decrease in G_0_/G_1_ phases, more pronounced in the soluble extract of green coffee (*p* < 0.05), in a dose-dependent manner. This effect was followed by S-phase arrest, mainly after treatment with green and light roasted Arabica coffee extracts (Table 2). Medium roasted extract promoted slight changes in cell cycle profile only in the S phase, while the dark roasted extract caused a reduction in the S phase.

**Table 2:** Effects of green and roasted Arabica coffee bean extracts (5000 and 10 000 μg mL^−1^) on the cell cycle progression in human prostate cancer PC-3 cells after 24 h of treatment

Sample	Cell cycle phase	CT	5000 μg ml^−1^	10 000 μg ml^−1^
**GC**	G_0_/G_1_	66.82 ± 4.57^a^	52.32 ± 1.14^b^	40.13 ± 4.04^C^
	S	3.30 ± 1.80^a^	30.96 ± 0.57^b^	41.62 ± 1.32^C^
	G_2_/M	21.02 ± 1.19^a^	16.72 ± 1.02^ab^	15.42 ± 41.62^b^
**LRC**	G_0_/G_1_	61.81 ± 2.52^a^	55.53 ± 1.51^b^	54.85 ± 0.96^b^
	S	3.88 ± 0.98^a^	8.67 ± 3.21^a^	15.74 ± 4.14^b^
	G_2_/M	20.12 ± 2.48^a^	9.42 ± 1.74^b^	17.83 ± 2.42^a^
**MRC**	G_0_/G_1_	62.23 ± 4.31^a^	62.73 ± 1.19^a^	61.90 ± 0.10^a^
	S	5.81 ± 0.52^a^	8.15 ± 0.39^b^	8.40 ± 0.56^b^
	G_2_/M	27.10 ± 3.67^a^	21.95 ± 1.60^b^	23.43 ± 1.32^ab^
**DRC**	G_0_/G_1_	64.07 ± 1.10^a^	60.21 ± 1.85^b^	61.08 ± 1.17^ab^
	S	9.82 ± 0.63^a^	7.93 ± 0.44^b^	8.30 ± 0.32^b^
	G_2_/M	22.63 ± 0.51^a^	21.88 ± 1.18^a^	22.78 ± 0.78^a^

Emerging evidence indicates that epigenetic and non-epigenetic mechanisms cause alteration of DNA methylation and loss of oestrogen receptors during tumour cycle progression, which in turn may be modifiable by dietary and lifestyle factors. It has also been shown that exposure to caffeine and caffeic acid significantly suppressed the proliferation of both ER+ (by 59% and 38%, respectively) and ER- (by 47% and 28%, respectively) human breast cancer cells (MCF-7 and MDA-MB-231) and inhibited DNA methylation.^67,68^

Previous studies have reported that caffeine caused an increase in the G_0_/G_1_ phase of mouse epithelial cells.^42,66^ and some studies have shown that hydroxycinnamic acids cause reduction of cell viability and apoptosis of tumour cells.^46,47^ Specifically, chlorogenic acids and their derivatives have been shown to be able to decrease the number of cells in phases G_0_/G_1_ and increase cells in phase S.^69,70^ This may explain the observation that green and light roasts caused greater increase in the S phase than did medium roast, because these compounds are degraded during roasting. Probably in dark roast, the amount of chlorogenic acids was not sufficient to have biological effects on cells.^27,62,65^

Many studies have shown the effects of green coffee extracts on normal cells and tumour cells, but few works have demonstrated and tested the effects of roasted coffee extracts on the viability of cancer cell lines.^27^

The development of cancer is associated with disorders in the regulation of the cell cycle. An S-phase checkpoint checks the fidelity of DNA replication and confirms there are no areas of unrepaired DNA damage from previous phases in the cell cycle. The DNA damage checkpoint arrests cells in the S phase depending on the cell cycle phase at the time damage was incurred. Checkpoints can also cause DNA repair and regulate cancer progression in addition to inducing cell cycle arrest.^71^ Our findings showed a cell cycle arrest in S phase by GC and LC. This fact may have promoted repair, or in the case of irreparable damage, stimulated cell death.

**Fig. 6:**
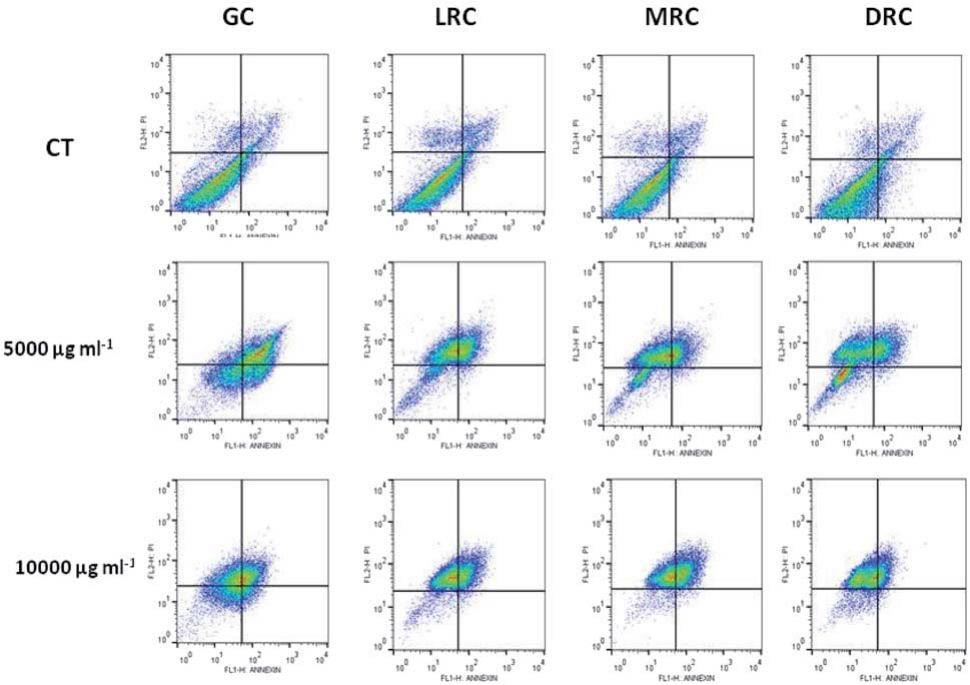
Effect of green and roasted Arabica coffee bean extracts on the rate of apoptosis in PC-3 cells after 24 h of incubation. Flow cytometry analysis of coffee extracts is illustrated. Different letters mean significant differences among treatments (*p* < 0.05). Abbreviations: GC – Green Coffee, LRC – Light Roasted Coffee, MRC – Medium Roasted Coffee, DRC – Dark Roasted Coffee.

To measure the rate of apoptosis, cells treated with the soluble coffee extracts showed a population shift to the upper left quadrant of the graph, as can be seen in Fig. 6. There was also displacement to the lower and upper right quadrants, representing the cell populations in early apoptosis and late apoptosis or non-apoptotic cell death. Medium and dark extracts had the capacity to alter cell populations, but also promoted changes in the non-apoptotic cells that were higher than those observed in the control, suggesting a potential toxicity effect. Apoptosis increased when we used the coffee extract concentration of 5000 and 10 000 μg mL^−1^ to treat the cells and the light roasted and green bean extracts were better than medium and dark extracts (Fig. 6).

Table 3 reports the percentages of cells of the control and the samples treated with the coffee extract concentrations of 5000 and 10 000 μg mL^−1^ for each phase of cell death.

**Table 3:** Effect of freeze-dried coffee extracts (5000 and 10 000 μg mL^−1^) on programmed cell death in a human prostate cancer cell line (PC-3) after 24 h of treatment^a^

Sample	Phases of cell death process	CT	5000 μg mL^−1^	10 000 μg mL^−1^
**GC**	Viable cells	91.90 ± 1.41^a^	38.08 ± 3.59^b^	55.85 ± 10.64^c^
	Early apoptosis	1.70 ± 0.42^a^	36.33 ± 11.18^b^	22.88 ± 11.42^ab^
	Late apop/necrosis	1.56 ± 0.43^a^	23.52 ± 9.89^b^	11.05 ± 4.22^ab^
	Non-apoptotic cells	4.86 ± 0.64^a^	2.18 ± 1.74^a^	10.23 ± 6.13^b^
**MRC**	Viable cells	91.93 ± 1.41^a^	75.07 ± 3.94^b^	66.03 ± 3.41^c^
	Early apoptosis	1.77 ± 0.42^a^	13.97 ± 3.52^b^	18.45 ± 2.36^b^
	Late apop/necrosis	1.56 ± 0.43^a^	5.78 ± 1.47^b^	8.76 ± 2.21^b^
	Non-apoptotic cells	4.86 ± 0.64^a^	5.18 ± 0.93^a^	6.77 ± 2.65^a^
**LRC**	Viable cells	91.98 ± 1.41^a^	66.98 ± 4.07^b^	68.25 ± 4.81^b^
	Early apoptosis	1.71 ± 0.42^a^	16.18 ± 2.83 ^b^	13.46 ± 2.99^b^
	Late apop/necrosis	1.56 ± 0.43^a^	8.03 ± 1.73^b^	8.66 ± 2.08^b^
	Non-apoptotic cells	4.86 ± 0.64^a^	8.79 ± 1.06^b^	9.64 ± 3.14^b^
**DRC**	Viable cells	91.99 ± 1.41^a^	75.37 ± 1.04^a^	79.56 ± 2.47^a^
	Early apoptosis	1.75 ± 0.42^a^	9.23 ± 0.88^b^	3.21 ± 0.53^c^
	Late apop/necrosis	1.56 ± 0.43^a^	6.26 ± 0.29^b^	1.89 ± 0.51^a^
	Non-apoptotic cells	4.86 ± 0.64^a^	9.12 ± 1.54^b^	9.36 ± 1.72^b^

a Results are expressed as the percentage of total cells. The data represent mean ± standard deviation values of triplicate experiments. Tukey test: (a–c) different letters in the same phase of death process and extract means that samples are different (*p* < 0.05) and same letters indicate samples are the same (*p* > 0.05). Abbreviations: GC – Green Coffee, LRC – Light Roasted Coffee, MRC – Medium Roasted Coffee, DRC – Dark Roasted Coffee. Viable cells (Annexin V−/PI−), early apoptosis (Annexin V+/PI−), late apoptosis/necrosis (Annexin V+/PI+), non-apoptotic cells (Annexin V−/PI+). PI – propidium iodide.

Cancer can occur when the balance between cell growth and death is disturbed. A cancer therapy that acts solely by induction of apoptosis without intrinsic cytotoxicity would be likely to cause the death of more normal cells than cancer cells. Our data indicate that green and light roasted extracts increased early and late apoptosis, with no significant changes in non-apoptotic cells.

These results might be directly related to the production of monomeric compounds of chlorogenic acids and caffeic acid, which modulate the cell's epigenetic activities by increasing DNA methylation and silencing some genes involved in proliferation. Even with the presence of acrylamide in its composition, the consumption of roasted coffee has strong effects on the prevention and treatment of chronic NCDs, including cancer, cardiovascular diseases, Parkinson's disease, Alzheimer's disease, among others.^27^

Several studies have shown no harmful effects on human health due to coffee consumption and some epidemiological studies have demonstrated significant positive effects of 3 to 4 cups of coffee per day in combating NCDs.^72^ Studies of bioavailability reveal high absorption and high permanence of caffeine in the bloodstream, a supposed passive transport absorbance of monomers of chlorogenic acids and an absorbance by active transport of caffeic acid in the colon.^73^

The literature has also reported that coffee extracts protect the living organism from various effects, including antioxidant activity, increased enzymatic activity of glutathione synthesis, and modulated oncogene expression.^65,74,75^ Similar to our research, it has been shown that coffee extracts showed cytotoxic activity, induced cell cycle arrest and induced apoptosis in Hela cells.^76^ The possible pathways through which coffee mediated biological activity can be correlated with the promotion of programmed cell death (apoptosis), cell cycle regulation and increased pro-apoptotic activity through the pathway involving caspases, bax and p53.^77^ Many bioactive compounds in Arabica coffee are well known, but many other compounds have not been described yet. The Maillard reaction by-products and its final products, melanoidins, are examples of this. Thus, further experiments are needed to elucidate the function of coffee's chemical substances on tumour cells.

## 4. Conclusions

Regular roasting (light to medium) causes significant positive changes in the chemical composition of coffee bean extracts, but darker roasting may negatively affect this composition and potential bioactivity, leading to chlorogenic acid breakdown and the formation of products, which may alter the health benefits.

The extraction of coffee solutions in a high-power ultrasonic device and the subsequent spray drying alone can generate a product with health appeal, although further studies are needed for confirmation. Our research demonstrates an important antiproliferative effect of coffee extract in human prostate cancer cell lines that can be contributes to cancer prevention.

## Conflicts of interest

There are no conflicts to declare.

## Supplementary Material
